# Quantifying symmetry in mandibular condyle motion: a real-time MRI approach

**DOI:** 10.22514/jofph.2025.079

**Published:** 2025-12-12

**Authors:** Justine Leclère, Karyna Isaieva, Guillaume Drouot, Romain Gillet, Jacques Felblinger, Pierre-André Vuissoz, Xavier Dubernard

**Affiliations:** ^1^IADI U1254, Inserm, University of Lorraine, 54000 Nancy, France; ^2^Oral Medicine Department, University Hospital of Reims, 51100 Reims, France; ^3^CIC 1433, Technical Innovation, Inserm, University of Lorraine, University Hospital of Nancy, 54000 Nancy, France; ^4^Guilloz Imaging Department, University Hospital of Nancy, 54000 Nancy, France; ^5^ENT Department, University Hospital of Reims, 51100 Reims, France

**Keywords:** Temporomandibular joint, Condylar kinematics, Mandibular condyle, Medical imaging, Detection algorithm

## Abstract

**Background**: The temporomandibular joints (TMJs) are essential for daily function and must operate in synergy to ensure optimal jaw movement. Real-time magnetic resonance imaging (RT-MRI) enables direct visualization of mandibular condyle motion; however, its application for symmetry assessment remains insufficiently studied. This exploratory study focuses on the consistency of condylar motion symmetry assessments, including visual evaluation of raw image series and 3D trajectories calculated from RT-MRI, as well as automatically extracted quantitative parameters. Given that well-correlated parameters are more likely to be reliable, this work aims to identify optimal methods for assessing symmetry of the condylar pathway using RT-MRI. **Methods**: The study includes 18 volunteers. A 2D real-time fast low angle shot (FLASH) sequence was used to acquire two sagittal and one axial planes. For quantitative analysis, mandibular condyles were segmented using a neural network. The extracted symmetry parameters were maximum displacement difference and maximum difference of amplitudes for spatial assessments, and latency and velocity peak delay for temporal evaluations. 3D trajectories were automatically generated using a previously validated method. Qualitative scoring of raw image series was conducted by two experts, and the 3D trajectories were evaluated by a dental surgeon. The agreement between qualitative scores was assessed using Cohen’s kappa test, while correlations among quantitative parameters were analyzed using Spearman’s test. **Results**: Results showed higher agreement in the axial plane (intra-observer κ = 0.68; inter-observer κ = 0.44) than in the sagittal plane (κ = 0.51 and 0.20, respectively). Inter-planar correlations were weak to moderate (ρ = 0.21–0.48), with latency showing the strongest correlation. **Conclusions**: For our dataset, latency emerged as the most robust temporal parameter. Additionally, motion analysis in the axial plane demonstrated greater consistency in both quantitative measurements and visual scoring. These findings suggest that the axial plane may be preferable for assessing motion symmetry in RT-MRI. **Clinical Trial Registration**: “METHODO” ClinicalTrials.gov Identifier: NCT02887053, approval: CPP EST-III, 08.10.01; “EDEN” ClinicalTrials.gov Identifier: NCT05218460, approval: CPP SUD-EST IV, 26.07.21.

## 1. Introduction

The temporomandibular joints (TMJs) are essential for chewing, speech, and swallowing. Subjected to constant mechanical forces, they must adapt continuously. Furthermore, the TMJs cannot move independently, so their motions are closely linked and must occur in synergy with a certain degree of symmetry to ensure optimal jaw function. This is why studying mandibular kinematics is important. However, mandibular motion remains difficult to capture precisely [1].

TMJ function is typically assessed indirectly through visual observation of mandibular motion [2]. To improve this evaluation, two main approaches have emerged: radiological imaging to assess joint integrity and extraoral dynamic tracking devices, such as jaw motion analyzers, used to study TMJ’s kinematics. However, each method has its drawbacks [3, 4, 5, 6]. Radiological imaging offers only static measurements and limited clinical accuracy [7, 8]. Tracking devices, while enabling dynamic analysis, rely on external markers to estimate TMJ position, often producing inconsistent data that require an experienced clinician and can sometimes produce false positive diagnosis [1, 3, 9]. Despite their advantages, neither technique provides a fully accurate representation of TMJ motion [10, 11].

Recent advancements in magnetic resonance imaging (MRI) allowed elimination of past limitations by improving dynamic imaging capabilities. The emergence of pseudo-dynamic imaging or cine-MRI has finally led to the development of real-time MRI (RT-MRI) [12, 13, 14]. With excellent temporal resolution and adequate spatial resolution RT-MRI allows simultaneous evaluation of TMJ structure and function thanks to a direct visualization of TMJs during their motion [15, 16]. This new way of studying the TMJ’s behaviors is experiencing a growing interest in both radiological and clinical fields [17].

Mandibular condyles are typically imaged in the sagittal plane, but in our previous study, we proposed using the axial plane for easier processing and simultaneous condyle imaging [18, 19]. Additionally, we introduced quantitative parameters for evaluating condylar motion symmetry [20]. Our next study enabled the automatic extraction of condylar trajectories from sagittal slices, which were combined with axial slices to produce 3D trajectories [21]. These advancements allow for evaluating motion symmetry from multiple perspectives: quantitative parameters derived from axial or sagittal planes, qualitative scores from video in both planes, or visual analysis of the 3D trajectories.

This exploratory study focuses on the consistency of condylar motion assessments. We compare condylar pathway symmetry, visually assessed by two experts from raw MRI dynamic series, with 3D trajectories and evaluate the intra- and inter-observer agreement in visual scoring. We also compare spatial and temporal symmetry parameters calculated from RT-MRI image series in both sagittal and axial planes. While any proposed criterion can be considered the gold standard, well-correlated parameters are likely to be more reliable. Therefore, this work aims to identify optimal methods that can be used for assessing the condylar pathway from RT-MRI.

## 2. Materials and methods

### 2.1 Volunteers and jaw motion task

This monocentric prospective study was conducted at the Regional and University Hospital Center of Nancy between January 2021 and November 2023. The data was acquired under two approved ethical protocols: “METHODO” (ClinicalTrials.gov Identifier: NCT02887053, approval: CPP EST-III, 08.10.01) and its successor “EDEN” (ClinicalTrials.gov Identifier: NCT05218460, approval: CPP SUD-EST IV, 26.07.21). This study was performed in line with the principles of the Declaration of Helsinki. The subjects’ rights have been protected by an appropriate Institutional Review Board and written informed consent was granted from all subjects.

A total of 18 adult volunteers (11 female, 7 male) were included, with ages ranging from 18 to 63 years (mean: 37.4 years). Participants, referred to as S1–S18, reported no history of orofacial pain during the inclusion interview. Exclusion criteria included general pathologies, particularly musculoskeletal diseases, as well as a painful TMJ or any head and neck invasive intervention. Individuals with posterior tooth loss involving five or more teeth (excluding wisdom teeth) were also excluded to ensure a proper mandibular stability. All participants provided informed consent before enrollment.

The volunteers were instructed by Expert 1 (JL) to perform slow jaw opening and closing movements (of 5 seconds each). Then they were asked to do successive and repeated cycles during the acquisition. Subjects were asked to reach their maximum possible amplitude for each movement.

### 2.2 MRI acquisitions

Real-time MR (Magnetic Resonance) images were acquired using a 3T MRI scanner (Prisma, Siemens Healthcare, Erlangen, BA, Germany) with a 64-channel head and neck coil (Siemens Healthcare, Erlangen, BA, Germany). All scans were performed with subjects in the supine position. The region of interest included the orofacial area, with both TMJs imaged simultaneously in each orientation. Imaging was performed using a radial Radio Frequency-Spoiled FLASH (RF-spoiled FLASH) sequence with T1 contrast in sagittal and axial planes, with 700 images in each series. Major MRI parameters are summarized in this manuscript, and further details are provided in our previous work [21].

#### 2.2.1 Sagittal plane real-time MRI acquisitions

For acquisitions in the sagittal plane, both TMJs were imaged quasi-simultaneously (by alternating slice) to allow direct comparison of their motion. The field of view (FoV) was 100 × 100 mm. To align with the global mandibular motion axis, the acquisition plane was defined from the most posterior point of the condyle to the external edge of the cortical bone at the level of the homolateral canine alveolus, following the lateral aspect of the ramus. The acquisition parameters were Repetition Time = 2.50 ms, Echo Time = 1.35 ms, slice thickness = 6 mm, and spatial resolution = 0.36 mm^2^. A total of 21 radial spokes were used, yielding a temporal resolution of 51 ms per image.

#### 2.2.2 Axial plane real-time MRI acquisitions

The axial plane was parallel to the Frankfurt Horizontal Plane (FHP), following a line connecting the uppermost points of both condyles in the coronal view. The acquisition plane was positioned below the level of the temporal eminence to capture condylar displacement throughout the movement. The sequence parameters were TR = 2.34 ms, TE = 1.47 ms, slice thickness = 8 mm. spatial resolution = 1.41 mm, FoV = 192 × 192 mm. The number of radial spokes was 9 and the temporal resolution = 21 ms per image. Two parallel planes with no gap between them were imaged simultaneously, and the slice with the clearest condyle visualization was selected for further analysis.

### 2.3 Post-processing and quantitative analysis

A k-means clustering, presented in [19], was applied to select 20 images per imaging series (two sagittal and one axial plane), which were then manually segmented by Expert 1 (JL, a dental surgeon specializing in temporomandibular disorders (TMD) care). In the axial plane, contours were traced along the cortical bone of the condylar head (Fig. 1A), while in the sagittal plane, they extended from the anterior to the posterior part, terminating below the condylar head at the start of the neck (Fig. 1B). All segmentations were performed using the Fiji software [22].

**Fig. 1.** MRI visualization of manually segmented mandibular condylar heads with anatomical reference planes. (A) Axial image showing the manually segmented right and left condylar heads (yellow) and the intersection of the mid-sagittal reference plane with the axial image (red line). (B) Sagittal image with the manually segmented condylar head (yellow) and the intersection of the Frankfurt horizontal plane with the sagittal image (red line).

A U-Net-based convolutional neural network, with a Dice score of 0.84 ± 0.07 in the axial plane and of 0.89 ± 0.04 in sagittal plane, had been previously validated for our dataset in [21]. This was then applied to automate segmentation. The centers of mass of the automatically segmented condyles were post-processed as described in [19] to smooth trajectory curves and identify opening and closing phases. For quantitative symmetry evaluation, mandibular motion was described in both spatial and temporal terms.

#### 2.3.1 Temporal parameters

Temporal parameters included latency (L) and velocity peak delay (VPD) between the right and left TMJs. Latency refers to the time interval between the start of the opening or closing movement of the right and left condyles. VPD represents the time delay between the two TMJs at their maximum velocity during jaw motion. Both temporal parameters were normalized to the total opening/closing duration.

#### 2.3.2 Spatial parameters

Spatial parameters included maximal difference of amplitudes (MDA) and maximal displacement difference (MDD). MDA quantifies the difference in maximum motion amplitudes reached by each condyle independently, measured in millimeters. MDD measures the largest distance between the right and left TMJs during their motion. This value was normalized by the maximum motion amplitude observed across the entire image series.

These parameters were calculated by projecting the measurements onto the Frankfurt Horizontal Plane (FHP) in sagittal images and onto the mid-sagittal plane in axial images. A more detailed description of the quantitative parameters calculation is provided in our previous work [19].

The axial and sagittal 2D trajectories were projected onto a 3D coordinate system as described in our previous article to extract 3D condylar trajectories [21]. These projections enabled a visual and quantitative assessment of the condylar motion.

### 2.4 Qualitative symmetry evaluation

#### 2.4.1 Direct visual evaluation

Two clinicians (Expert 1: JL, a dental surgeon with expertise in TMD care, and Expert 2: RG, a radiologist specializing in musculoskeletal imaging) were asked to visually assess condylar trajectories (JL_1_ and RG) by independently reviewing raw real-time MRI videos for the axial and sagittal planes and for both opening and closing movements. In the absence of pathological cases, the decision was made to limit the scoring to a binary scoring system (Score 0 or 1) to reflect the overall impression of movement symmetry. A score of 0 was assigned if the condylar trajectory appeared uniform and symmetric (with no obstructions or visual deviations), while a score of 1 was given if the trajectory was irregular, with visible asymmetry or deviations. When assigning a score of 1, experts were also required to identify the lagging TMJ. To evaluate intra-observer variability, Expert 1 (JL_1_) scored the same videos again 21 days later (JL_2_). Prior to scoring, the videos were independently randomized for each expert in both planes eliminate bias in the evaluation.

#### 2.4.2 Three-dimensional condylar trajectory assessment

The 3D condylar trajectory was assessed for symmetry between the right and left TMJs by Expert 1. A score of 0 was assigned if the trajectory difference was less than 1 mm across all three planes. If the amplitude difference exceeded 1 mm in at least two planes, the trajectory was scored 1, and the TMJ with the smaller amplitude was identified as the lagging side.

### 2.5 Statistical analysis

To evaluate the consistency of visual assessments, expert scores in the sagittal and axial planes were compared using the weighted Cohen’s Kappa test, assessing both intra-observer (JL_1_, JL_2_) and inter-observer (JL_1_, RG) agreement. The same test was used to compare mean expert scores (JL_1_ + RG) in each plane with 3D condylar trajectory scores. The kappa-values were interpreted according to McHugh ML [23].

To analyze correlations between quantitative parameters, Spearman’s correlation coefficient was calculated for inter-plane (axial (AX) and sagittal (SAG)) comparisons (MDD_AX_ *vs.* MDD_SAG_; MDA_AX_ *vs.* MDA_SAG_; L_AX_ *vs.* L_SAG_; VPD_AX_ *vs.* VPD_SAG_); and intra-plane relationships (MDD *vs.* MDA; VPD *vs.* L; MDD *vs.* VPD; MDD *vs.* L; MDA *vs.* VPD and MDA *vs.* L). The interpretation of Spearman’s rho values followed Schober P *et al*. [24].

## 3. Results

Four subjects were excluded from the study (S2, S4, S6, S18), as previously documented in [21]. The primary reason was head motion, which caused misalignment between the axial and sagittal plane positions. Additionally, for one participant (S18), jaw movement was too slow, resulting in an incomplete motion cycle during the acquisition. The final study cohort consisted of 14 subjects (9 women and 5 men) with a mean age of 38 years.

### 3.1 Qualitative scores consistency

The visual scores from both experts are provided in the **Supplementary material**. Fig. 2 presents examples of trajectories extracted through post-processing and classified as 0 or 1 based on direct assessment.

**Fig. 2.** MR images and automatically extracted condylar pathways illustrating expert visual assessments of unprocessed image series. Trajectories are displayed in the axial plane (left) and in the sagittal plane for the left TMJ (center) and right TMJ (right). Color encoding represents time progression during mouth opening, with dark blue indicating the onset and brown marking the end of the movement. (A) Symmetric condylar trajectory during opening (scored 0 by experts). (B) Visually asymmetric condylar pathways during opening (scored 1 by experts).

The weighted Cohen’s Kappa value for intra-observer agreement (JL_1_ *vs.* JL_2_) was 0.68 in the axial plane (substantial agreement) and 0.51 in the sagittal plane (moderate agreement). Inter-observer agreement (JL_1_ *vs.* RG) was moderate in the axial plane (κ_AX_ = 0.44) and slight in the sagittal plane (κ_SAG_ = 0.20). These results indicate that scoring in the axial plane was more consistent than in the sagittal plane for both intra-observer and inter-observer comparisons.

Comparison of mean visual scores with three-dimensional condylar trajectories (3DTC) revealed moderate agreement in the axial plane for the opening movement (κ = 0.43) and slight agreement for the closing movement (κ = 0.19). In the sagittal plane, agreement was lower, with κ = 0.28 for opening and κ = 0.14 for closing. Overall, opening movements showed better agreement than closing movements, with the axial plane offering greater scoring consistency.

### 3.2 Quantitative parameters consistency

#### 3.2.1 Correlation between different plane orientations

The mean L_AX_ was 0.14, ranging from 0 (subjects S11, S15) to a maximum of 0.55 (S13). The mean L_SAG_ was also 0.14, with values ranging from 0.01 (S13) to 0.37 (S8). A moderate positive correlation was observed between the two planes (ρ = 0.48).

The mean VPD_AX_ was 0.10, ranging from no relative delay (S7, S8, S10) to a maximum of 0.30 (S1). The mean VPD_SAG_ was 0.09, with values ranging from no relative delay (S1, S14, S16) to a maximum of 0.27 (S7). The correlation between the two planes was weakly positive (ρ = 0.33).

The mean MDD_AX_ was 2.37, ranging from 0.13 (S8) to 11.11 (S3). The mean MDD_SAG_ was 2.30, with values ranging from 0.07 mm (S15) to 9.26 mm (S3). Despite similar mean values, the correlation between the two planes was weakly positive (ρ = 0.21).

The mean MDA_AX_ was 1.94 mm, ranging from 0.24 mm (S12) to 10.98 mm (S3). The mean MDA_SAG_ was 2.10 mm, with values between 0.65 mm (S14) and 9.26 mm (S3). The correlation between planes was weakly positive (ρ = 0.28). Additionally, the subject showing the greatest asymmetry (S2) demonstrated elevated MDA values in both planes.

#### 3.2.2 Correlation between different parameters

The correlation between different parameters is presented in Fig. 3.

**Fig. 3.** Spearman correlation matrix between study parameters: Velocity Peak Delay (VPD), Latency (L), Maximal Displacement Difference (MDD), and Maximal Difference of Amplitudes (MDA). AX: axial plane; SAG: sagittal plane; op: opening; cl: closing.

The temporal parameters L and VPD were weakly-to-moderately correlated between them in both planes, with ρ = 0.34 in the axial plane and ρ = 0.15 in the sagittal plane. The spatial parameters MDD and MDA were stronger correlated, with ρ_opening_ = 0.65 and ρ_closing_ = 0.74 for the axial plane and ρ_opening_ = 0.92 and ρ_closing_ = 0.75 for the sagittal plane.

Regarding the correlation between spatial and temporal parameters, in the axial plane, the strongest correlation was observed between L and MDA, particularly during closing movements (ρ_closing_ = 0.48). This was higher than the correlation between L and MDA during opening movements (ρ_opening_ = 0.23) and between L and MDD (ρ = 0.29). In the sagittal plane, L correlated more strongly with MDA during opening movements (ρ_opening_ = 0.27) than during closing movements (ρ_closing_ = 0.10) or with MDD (ρ = 0.24). The correlation between VPD and spatial parameters was weakly positive in the sagittal plane with MDA (ρ = 0.39) but minimal or even negative for all other spatial parameters.

These findings indicate that latency (L) is more strongly correlated with spatial parameters than VPD.

## 4. Discussion

This exploratory prospective study demonstrates that real-time MRI can effectively capture temporomandibular joint (TMJ) kinematics using both qualitative and quantitative parameters. The acquired images were successfully post-processed to derive a variety of metrics, with the semi-automated segmentation using a deep learning neural network proving to be both efficient and precise [19]. The combination of MRI data with automated measurements further supports the potential use of machine learning to improve clinical assessments [25, 26].

The qualitative evaluation demonstrated moderate agreement between the experts. This variability likely reflects the inherent challenges in visually assessing dynamic joint motion in real time. Human visual perception has known limitations in tracking distant points within the same image and comparing side-by-side [27]. In particular, faster closing movements, compared to opening movements, present additional difficulties for consistent evaluation. This acceleration can be explained by the activation of stronger elevator muscles (temporalis and masseter) during closing movement while the opening relies on weaker suprahyoid muscles [28]. Greater training, the ability to slow down image sequences, and a more detailed scoring scale for TMJ asymmetries could improve the reliability of visual assessments.

This study introduces quantitative parameters that describe the symmetry of TMJ movements, in terms of both spatial and temporal dimensions. Processing MR images in this way presents a promising approach to studying mandibular kinematics and may become a valuable tool for clinicians [20, 29, 30]. The spatial parameters were highly correlated, as could be expected. Temporal parameters, on the other hand, exhibit only slight correlations with each other, both independently and across planes. The probable explanation of this difference is that selected spatial parameters are directly derived from distance measurements. In contrast, the calculation of temporal parameters required certain assumptions. Specifically, velocity extraction involved post-processing with filtering, and the latency estimation relied on a threshold set at 10% of the maximum velocity, which may introduce additional variability.

The combination of visual scores with 3DTC achieves a slight to moderate agreement depending on the plane studied. This may be explained by the need to acquire axial and sagittal sequences separately to maintain adequate image quality. While mandibular motion patterns remain largely comparable within individuals, minor variations occur between series of movements, and even within the same series. These variations can be observed from different series of movement but even into the same series [31, 32]. These differences may result from masticatory muscle fatigue, anatomical factors, age and sex [33, 34].

Certain limitations should be considered in this study. The relatively small sample of asymptomatic volunteers (n = 18) was further reduced to 14 due to acquisition plane misalignment and MRI acquisition failures. Providing more precise guidelines for acquisition plane positioning and anatomical landmark identification could help minimize these errors. Since these acquisition protocols are not routinely performed by MRI technicians, additional training may be required to improve accuracy. A larger cohort would enable further validation of our method, the identification of outliers, and a more precise distinction between physiological and non-physiological TMJ movements [20]. Furthermore, qualitative agreement between the experts remains variable, and visual evaluation alone may not be sufficient to properly assess jaw motion symmetry. Despite these challenges, this study successfully demonstrates the feasibility of real-time MRI for TMJ motion analysis, with further methodological improvements expected in future work. Additionally, this approach enables the simultaneous anatomical and kinematic evaluation of both TMJs [35].

While previous studies have described aspects of condylar pathway behavior, accurate measurements remain limited, and no reference dataset exists for asymptomatic populations. This study provides the first comprehensive anatomical analysis of three-dimensional trajectories of mandibular condyles, for both opening and closing motions in asymptomatic individuals.

## 5. Conclusions

This study analyzed both qualitative and quantitative parameters to characterize mandibular motion, with a particular focus on asymmetry. By combining visual assessments with automated measurements, it provides a more comprehensive evaluation.

Among the temporal parameters, latency emerged as a globally more reliable metric than peak velocity delay, in terms of its correlation with spatial parameters and between different acquisition plane orientations. Additionally, for our dataset, motion analysis in the axial plane showed greater consistency in both measured parameters and visual scoring compared to the sagittal plane. These findings suggest that the axial plane likely provides more reliable measurements and may be the preferred choice for assessing motion symmetry in 2D dynamic MRI.

This work presents an original and novel framework for studying condylar trajectories, advancing the understanding of TMJ biomechanics. Even if other studies with a larger cohort and pathological cases will be needed to offer the possibility for an application in the clinical field, the presented study provides measurable parameters that may distinguish healthy from pathological motion. We hypothesize that affected individuals will show increased asymmetry, which remains to be tested.

## Supplementary Material

Supplementary material associated with this article can be found, in the online version, at https://files.jofph.com/ files/article/1999376360147959808/attachment/ Supplementary%20material.docx.


