# Supplementary material for: Quantifying symmetry in mandibular condyle motion: a real-time MRI approach
Source: J Oral Facial Pain Headache. 2025 Dec 12;39(4):227–34. doi: 10.22514/jofph.2025.079 (PMC12727175; doi:10.22514/jofph.2025.079)
Supplement: Supplementary file 1 [file Supplementary-material.docx]

Supplementary material

Supplementary Table 1. Expert cores assessing the visible asymmetry for each subject (S1 to S18) based on axial plane imaging. When asymmetry is observed, the delayed TMJ is specified.

| Subject | JL1 | | | | JL2 | | | | RG | | | |
| --- | --- | --- | --- | --- | --- | --- | --- | --- | --- | --- | --- | --- |
|  | Opening | | Closing | | Opening | | Closing | | Opening | | Closing | |
|  | Score | DT | Score | DT | Score | DT | Score | DT | Score | DT | Score | DT |
| S1 | 1 | R | 1 | R | 1 | R | 1 | R | 1 | R | 1 | L |
| S2 | 0 | — | 0 | — | 0 | — | 0 | — | 1 | L | 1 | R |
| S3 | 1 | R | 0 | — | 1 | R | 1 | R | 1 | R | 1 | L |
| S4 | 1 | R | 1 | L | 1 | R | 1 | L | 1 | R | 1 | L |
| S5 | 1 | L | 1 | R | 1 | L | 1 | L | 1 | L | 1 | R |
| S6 | NA | — | NA | — | 1 | R | 0 | — | NA | — | NA | — |
| S7 | 1 | R | 0 | — | 1 | R | 1 | R | 1 | L | 0 | — |
| S8 | 0 | — | 0 | — | 0 | — | 0 | — | 0 | — | 1 | L |
| S9 | 0 | — | 0 | — | 0 | — | 0 | — | 0 | — | 1 | L |
| S10 | 0 | — | 0 | — | 0 | — | 0 | — | 0 | — | 1 | L |
| S11 | 0 | — | 0 | — | 0 | — | 0 | — | 1 | L | 0 | — |
| S12 | 0 | — | 0 | — | 0 | — | 0 | — | 0 | — | 1 | L |
| S13 | 1 | R | 0 | — | 1 | R | 0 | — | 1 | R | 0 | — |
| S14 | 1 | L | 0 | — | 1 | L | 1 | R | 1 | L | 1 | R |
| S15 | 1 | L | 1 | R | 0 | — | 0 | — | 1 | R | 1 | L |
| S16 | 0 | — | 0 | — | 0 | — | 0 | — | 0 | — | 0 | — |
| S17 | 1 | R | 0 | — | 1 | R | 0 | — | 1 | R | 1 | L |
| S18 | 1 | L | 1 | R | 1 | L | 1 | R | 1 | L | 1 | R |

DT: Delayed TMJ; L: Left TMJ; R: Right TMJ; JL1: Expert 1, first scoring; JL2: Expert 1, second scoring; RG: Expert 2 scoring; NA: Non-applicable.

Supplementary Table 2. Expert cores assessing the visible asymmetry for each subject (S1 to S18) based on sagittal plane imaging. When asymmetry is observed, the delayed TMJ is specified.

| Subject | JL1 | | | | JL2 | | | | RG | | | |
| --- | --- | --- | --- | --- | --- | --- | --- | --- | --- | --- | --- | --- |
|  | Opening | | Closing | | Opening | | Closing | | Opening | | Closing | |
|  | Score | DT | Score | DT | Score | DT | Score | DT | Score | DT | Score | DT |
| S1 | 1 | R | 0 | — | 0 | — | 0 | — | 0 | — | 1 | L |
| S2 | 0 | — | 0 | — | 0 | — | 0 | — | 0 | — | 0 | — |
| S3 | 1 | R | 0 | — | 1 | L | 0 | — | 1 | L | 1 | R |
| S4 | 0 | — | 1 | L | 0 | — | 1 | L | 1 | L | 0 | — |
| S5 | 1 | R | 0 | — | 0 | — | 0 | — | 0 | — | 0 | — |
| S6 | 0 | — | 0 | — | 0 | — | 0 | — | 0 | — | 0 | — |
| S7 | 1 | R | 0 | — | 1 | R | 0 | — | 0 | — | 0 | — |
| S8 | 0 | — | 0 | — | 0 | — | 0 | — | 0 | — | 0 | — |
| S9 | 0 | — | 0 | — | 0 | — | 0 | — | 0 | — | 0 | — |
| S10 | 1 | R | 0 | — | 0 | — | 0 | — | 1 | L | 0 | — |
| S11 | 0 | — | 0 | — | 0 | — | 0 | — | 0 | — | 0 | — |
| S12 | 0 | — | 0 | — | 1 | R | 0 | — | 0 | — | 0 | — |
| S13 | 0 | — | 0 | — | 0 | — | 0 | — | 0 | — | 0 | — |
| S14 | 1 | R | 0 | — | 1 | R | 0 | — | 0 | — | 0 | — |
| S15 | 1 | R | 0 | — | 0 | — | 0 | — | 1 | L | 1 | R |
| S16 | 0 | — | 1 | L | 0 | — | 0 | — | 0 | — | 0 | — |
| S17 | 1 | R | 0 | — | 1 | R | 0 | — | 1 | L | 1 | R |
| S18 | 1 | R | 1 | L | 1 | R | 0 | — | 0 | — | 1 | L |

DT: Delayed TMJ; L: Left TMJ; R: Right TMJ; JL1: Expert 1, first scoring; JL2: Expert 1, second scoring; RG: Expert 2 scoring; NA: Non-applicable.
